# An iron-acquisition-deficient mutant of *Corynebacterium pseudotuberculosis* efficiently protects mice against challenge

**DOI:** 10.1186/1297-9716-45-28

**Published:** 2014-03-06

**Authors:** Dayana Ribeiro, Flávia de Souza Rocha, Kátia Morais Costa Leite, Siomar de Castro Soares, Artur Silva, Ricardo Wagner Dias Portela, Roberto Meyer, Anderson Miyoshi, Sérgio Costa Oliveira, Vasco Azevedo, Fernanda Alves Dorella

**Affiliations:** 1 Laboratório de Genética Celular e Molecular, Instituto de Ciências Biológicas, Universidade Federal de Minas Gerais, Av. Antônio Carlos, 6627 - Pampulha, CP 486, CEP 31, Belo Horizonte, MG 270-901, Brazil; 2 Instituto de Ciências Biológicas, Universidade Federal do Pará, Belém, PA, Brazil; 3 Instituto de Ciências da Saúde, Universidade Federal da Bahia, Salvador, BA, Brazil; 4 Laboratório de Imunologia de Doenças Infecciosas, Instituto de Ciências Biológicas, Universidade Federal de Minas Gerais, Av. Antônio Carlos, 6627 - Pampulha, CP 486, CEP 31, Belo Horizonte, MG 270-901, Brazil

## Abstract

Caseous lymphadenitis (CLA) is a chronic disease that affects sheep and goats worldwide, and its etiological agent is *Corynebacterium pseudotuberculosis*. Despite the economic losses caused by CLA, there is little information about the molecular mechanisms of bacterial pathogenesis, and current immune prophylaxis against infection has been unable to reduce the incidence of CLA in goats. Recently, 21 different mutant strains of *C. pseudotuberculosis* were identified by random mutagenesis. In this study, these previously generated mutants were used in mice vaccination trials to develop new immunogens against CLA. Based on this analysis, CZ171053, an iron-acquisition-deficient mutant strain, was selected. After challenge with a virulent strain, 80% of the animals that were immunized with the CZ171053 strain survived. Furthermore, this vaccination elicited both humoral and cellular responses. Intracellular survival of the bacterium was determined using murine J774 cells; in this assay, the CZ171053 had reduced intracellular viability. Because iron acquisition in intracellular bacteria is considered one of their most important virulence factors during infection, these results demonstrate the immunogenic potential of this mutant against CLA.

## Introduction

*Corynebacterium pseudotuberculosis* is a facultative gram-positive intracellular pathogen that is the etiological agent of caseous lymphadenitis (CLA) in sheep and goats. CLA is a chronic debilitating infection, and its main symptoms can be divided into external CLA, which affects superficial lymph nodes, and internal CLA, which leads to the development of abscesses in internal lymph nodes and organs, including the liver, lungs and kidneys [1-3]. This disease has a worldwide distribution and causes economic losses in several countries, principally to producers in developing countries such as Brazil, where sheep and goat breeding are of increasing economic importance [4-10].

Once established in a herd, CLA is difficult to treat because it is generally refractory to antibiotic therapy, which is also very expensive [11]. Therefore, several studies have been conducted to develop vaccines that can effectively control CLA. Although these vaccines are useful and offer reasonable protection, there are several problems associated with their use. All of these vaccines need to be administered at least twice, which contributes to the misuse of the vaccines by farmers and increases the cost. Furthermore, not all vaccines available for use in goats have the same efficacy in sheep, and they are not licensed in all countries [2,12]. Some experimental vaccines have been tested using different strategies such as vaccines using attenuated or inactivated bacteria, cell wall fractions and DNA [13-25]. These vaccines confer variable levels of protection, but their safety profiles remain questionable mainly because their side effects, which are more intense in goats. These side effects include the formation of lesions or abscesses at the injection site, fever, malaise and reduced milk production [26,27].

Driven by the paucity of information on the molecular basis of *C. pseudotuberculosis* virulence, we identified and characterized bacterial genes based on the cellular localization of their products, focusing mainly on those proteins that are anchored and secreted. To identify such genes, we used, for the first time in this species, a reporter transposon-based system named TnFuZ [28]. Using this tool, we identified 34 mutants exhibiting a blue phenotype from a repertoire of 1500 kanamycin-resistant mutants [3]. By sequencing the transposon insertion site in these mutants, we identified 21 different loci encoding fimbrial and transport subunits, hypothetical proteins and proteins with unknown-function in *C. pseudotuberculosis*. In this present study, we tested these mutants in immunization assays to assess their virulence potential and their capacity to protect mice against challenge. Furthermore, we studied the cytokine and immunoglobulin production induced by the best vaccine candidates.

## Materials and methods

### Bacterial strains and growth conditions

The following strains were used: the previously generated *C. pseudotuberculosis* TnFuZ recombinant strains [3], the T1 pathogenic wild-type parental strain and the caprine-pathogenic MIC-6 strain. The strains were grown aerobically in Brain Heart Infusion broth (BHI, Oxoid Ltd., Hampshire, England) at 37 °C. The mutant strains were grown in the presence of kanamycin (kanamycin sulphate, 25 μg/mL; solid and liquid media) and 5-bromo-4-chloro-3-indolylphosphate (BCIP, 40 μg/mL; Sigma-Aldrich Co., St. Louis, MO, USA; solid medium), a substrate that allows the recovery of *C. pseudotuberculosis* insertional mutant colonies with positive alkaline phosphatase activity.

### Animal model

One hundred fifty BALB/c mice between six and eight weeks of age, that were susceptible to *C. pseudotuberculosis* infection, were used in this assay. They were provided by the Animal Care Facility of the Biological Sciences Institute from the Federal University of Minas Gerais and were handled according to the guidelines of the UFMG Ethics Committee on Animal Testing.

### Immunization assay, challenge and assessment of protection level

The immunization schedule was as follows: twenty-two groups of five mice each were intraperitoneally inoculated with 2 × 10^6^ Colony forming unit (CFU) of each mutant strain (CZ171049 and CZ171061 with different transposon insertions), with was, the same dose used by Simmons et al. [23]. Another group of five animals received the T1 wild-type strain under the same conditions, and a 24^th^ group of five mice was inoculated with 100 μL of saline solution (0.9% NaCl). The mice were intraperitoneally challenged 21 days after immunization with 2 × 10^6^ C.F.U of the *C. pseudotuberculosis* MIC-6 virulent strain. The protection conferred by the immunization process was evaluated by comparing the survival of the immunized animals to those inoculated with the T1 wild-type strain and saline solution. The mice were evaluated for four weeks after challenge.

For the second immunization assay, three groups of ten mice each were inoculated with 2 × 10^6^ CFU of the CZ171053 strain, 2 × 10^6^ CFU of the T1 strain or 100 μL of saline solution. The immune protection was evaluated using the procedure described above.

### Detection of specific IgG, IgG1 and IgG2a antibodies

Serum samples were taken 21 days after immunization. Blood samples were collected through retro-orbital bleeding. After coagulation, the blood was centrifuged at 3000 rpm for ten minutes. The serum samples were collected and stored at −20 °C. These samples were analysed using an enzyme-linked immunosorbent assay (ELISA) to measure the total levels of specific IgG, IgG1 and IgG2a antibodies. The plates were incubated with 200 μL of the supernatant of a MIC-6*C. pseudotuberculosis* culture grown for 72 h (total protein concentration of 4 μg/mL); the plates were then incubated for 18 h at 4 °C. The wells were blocked with 0.1% Tween 20 and 10% fat-free milk powder in PBS (pH 7.4) for two hours at room temperature. The plates were then incubated with 100 μL of the serum samples, diluted 1:50 in PBS-Tween, for one hour at 37 °C. The wells were washed three times with PBS-Tween between incubations. Anti-mouse IgG conjugated to horseradish peroxidase (Promega, Madison, WI, USA) was diluted 1:10 000 in PBS-Tween and added to the wells, and the plates were incubated for one hour at 37 °C. The same procedure was performed with horseradish peroxidase-conjugated anti-mouse IgG1 (Promega) diluted 1:5000 and anti-mouse IgG2a (Promega) (diluted 1: 2000). The plates were developed with 200 pmol of o-phenylenediamine (Sigma) and 0.05% H_2_O_2,_ for ten minutes, and the reaction was stopped with 50 μL of 2 N H_2_SO_4_. After incubation for ten minutes at room temperature, the optical density at 492 nm (OD492) was measured.

### Cytokine analysis

For the cytokine analysis, five immunized mice from the CZ171053-, T1- and saline-infected groups were sacrificed before challenge, and their spleens were collected aseptically. Erythrocytes were removed by treatment with 0.017 M Tris-ammonium chloride, and the remaining leukocytes were washed twice with PBS (pH 7.4) and suspended in RPMI (RPMI-1640; Gibco BRL, Life technologies, Carlsbad, CA, USA) containing 10% fetal bovine serum, 100 U/mL penicillin and 100 μg/mL streptomycin. After adjusting the cellular concentrations to 5 × 10^6^ viable cells/mL in 100 μL, the samples were seeded in 96-well tissue culture plates. The cells were stimulated with the supernatant of a 72-h MIC-6*C. pseudotuberculosis* culture (protein concentration of 4 μg/mL) [29]. The positive control wells were stimulated with concanavalin A (2 μg/mL; Sigma), and culture medium was added to the negative control wells. The supernatants were collected 24 h after stimulation and used for IL-4, IL-10, IFN-γ and TNF-α quantification using commercial sandwich ELISA kits (Duo Set ELISA Development System, R&D Systems, Minneapolis, MN, USA), according to the manufacturer’s instructions.

### Determination of intracellular survival

J774 macrophage-like cells, derived from murine lymphoma were cultivated in Dulbecco’s modified Eagle essential medium (DMEM, Sigma) supplemented with 5% fetal bovine serum, 50 μg/mL gentamicin and 2.5 μg/mL fungizone at 37 °C in a 5% CO_2_ atmosphere. The CZ171053 mutant strain and T1 wild-type strain were grown for 48 h at 37 °C, washed three times with PBS, resuspended in DMEM to at a concentration of 10^6^ CFU/mL and used to infect J774 cells (10 bacteria: 1 cell). For the determination of intracellular viability, the infected J774 monolayers were washed six times with PBS and treated with 150 μg/mL gentamicin sulphate (Sigma) then diluted in DMEM for one hour after one, three and six hours of incubation. The cells were washed six times with PBS to eliminate the dead bacteria in the extracellular medium. The monolayers were lysed with 0.5 mL of 0.1% TritonX-100 (Sigma) in PBS, and the supernatant was plated on BHI agar. The number of intracellular bacteria was determined by counting the number of CFU.

### Statistical analysis

The results were expressed as the mean ± standard deviation. The differences among the experimental groups were calculated using one-way ANOVA followed by the Bonferroni (post-test levels of protection and immunoglobulin and cytokine production) or Tukey’s (intracellular viability) tests. The data were considered significantly different when *p* < 0.05. Both tests were performed using GraphPad Prism version 5.00 for Windows (GraphPad Software, San Diego, CA, USA) [30].

## Results

### Immunization assay, challenge and assessment of protection level

Using a transposon-based random mutagenesis system, 34 alkaline phosphatase-positive recombinant strains of *C. pseudotuberculosis* were obtained; in these strains transposon insertions were identified at 21 different loci. In this study, 22 of the mutant strains were tested in mice vaccination trials to select potential immunogenic candidates against caseous lymphadenitis.

**Figure 1:**
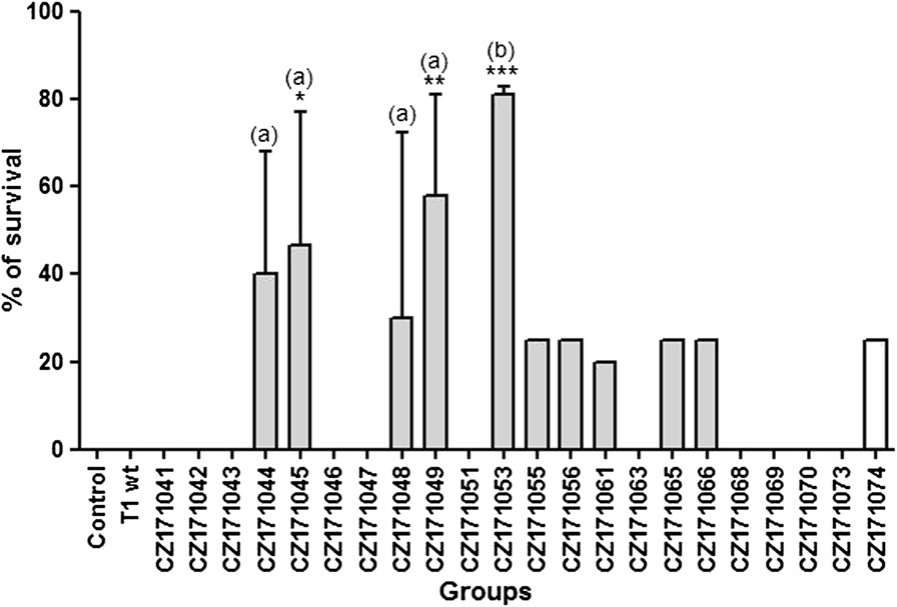
**Percentage of immunized animals protected from the experimental challenge with *C. pseudotuberculosis.*** Twenty-one days after immunization with recombinant strains of *C. pseudotuberculosis,* the animals were challenged with 10^6^ C.F.U. of the MIC-6 virulent wild-type strain. Protection was defined as the survival of the immunized mice for four weeks after the challenge. Bars marked with (a) and (b) correspond to the mutants tested three times and five times, respectively. *p < 0.05, **p < 0.01, ***p < 0.001 versus control (ANOVA, post-test Bonferroni).

Mice that were inoculated with the CZ171053 recombinant *C. pseudotuberculosis* strain, which is an iron-acquisition-defective mutant, showed the best protection level; 80% of the mice immunized with this strain survived after challenge with the virulent strain (Figure 1). The mice immunized with other mutants did not acquire a protection level above 60% (CZ171049). The animals immunized with the T1 wild-type strain did not survive long enough to be challenged; all of these mice died three days after immunization.

After selection of the CZ171053 strain as a potential immunogenic candidate, a new immunization assay was performed to evaluate the immunoglobulin- and cytokine-induction profiles after immunization. Three groups of ten mice received the CZ171053 mutant, T1 wild-type strain or 100 μL of saline solution according the immunization schedule described above. Serum samples and splenocytes were collected 21 days after immunization.

**Figure 2:**
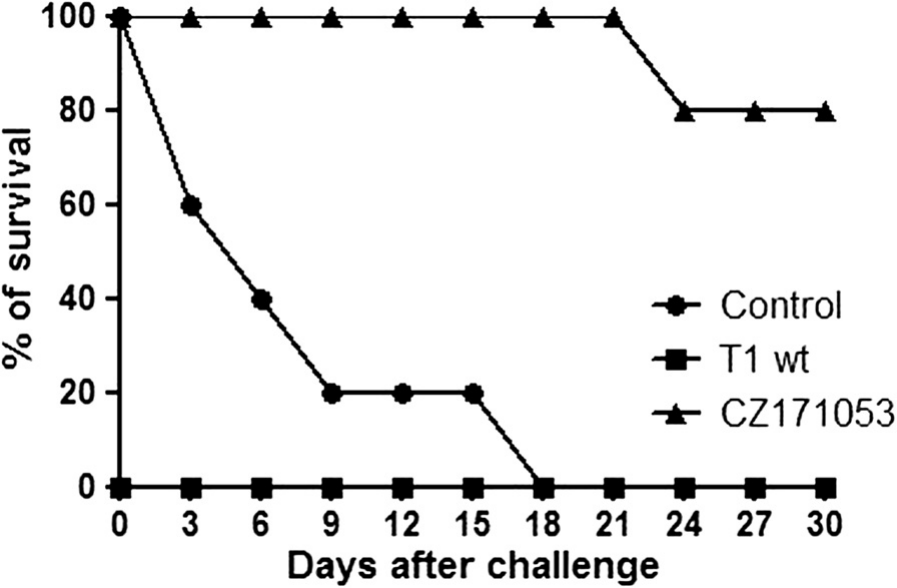
**Kinetics of the survival of animals immunized with the CZ171053 recombinant strain of *C. pseudotuberculosis*.** The lines show the daily percentage of survival of the immunized mice challenged with 10^6^ CFU of the MIC-6 virulent wild-type strain for four weeks post-infection. The data are from a representative experiment using ten mice per group.

In this second experiment, inoculation with the CZ171053 strain protected 80% of the MIC-6-challenged mice (Figure 2). The animals that were inoculated with the T1 wild-type strain did not survive until they were challenged with the MIC-6 strain, and all animals from the control group died within eighteen days of inoculation with the MIC-6 strain.

### Detection of specific IgG, IgG1 and IgG2a antibodies

**Figure 3:**
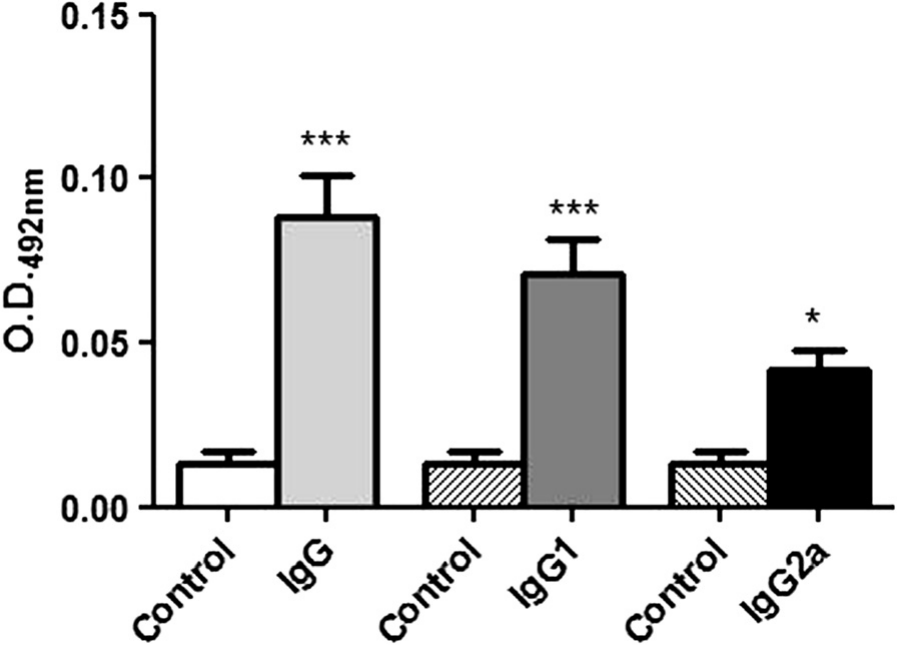
**Serological response of the mice following immunization with the CZ171053 recombinant strain of *C. pseudotuberculosis.*** The mice were bled 21 days after immunization, and the production of specific IgGs and their subtypes, IgG1 and IgG2a, was measured by ELISA analysis. The bars represent the mean ± standard deviation obtained in a representative experiment using ten mice per group. *p < 0.05, **p < 0.01, ***p < 0.001 versus control (ANOVA, post-test Bonferroni).

The serum samples from mice immunized with the CZ171053 strain were tested by ELISA 21 days after immunization to detect the production of specific IgG, IgG1 and IgG2a antibodies. IgG1 and IgG2a were investigated separately because IgG1 is related to a Th2 cellular immune response, whereas IgG2a is related to a Th1 response in the same species. The ELISA analysis revealed that mice immunized with the CZ171053 strain produced significantly higher levels of *C. pseudotuberculosis-*specific IgG, IgG1 and IgG2a than the control mice (Figure 3), indicating that the humoral immune response was activated by the immunization.

### Cytokine analysis

**Figure 4:**
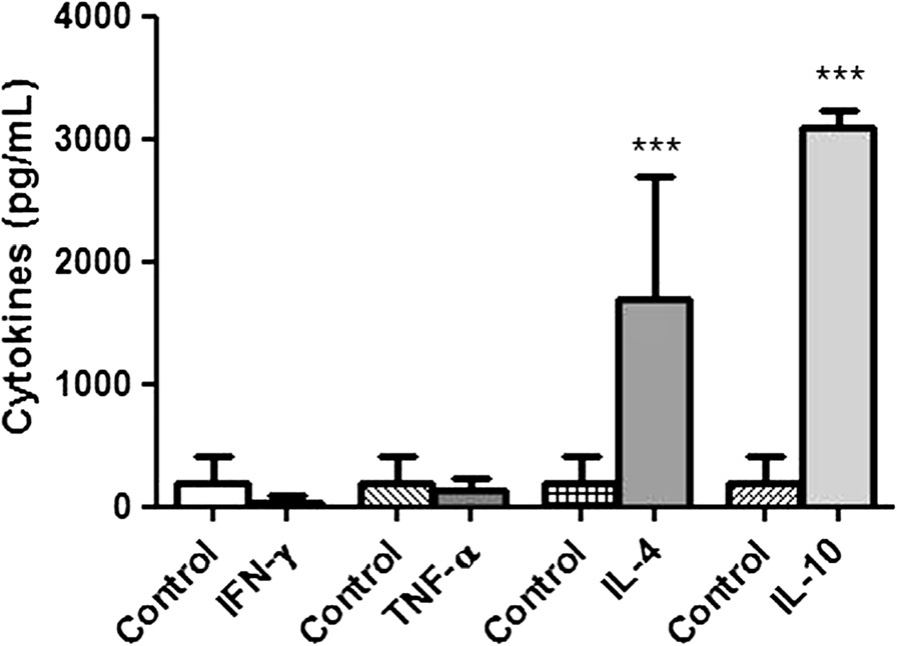
**Cytokine detection in the supernatant of cultured mouse splenocytes that were isolated and pooled from CZ171053-vaccinated mice.** The bars represent the mean ± standard deviation of a representative experiment using five mice per group. ***p < 0.001 versus control (ANOVA, post-test Bonferroni).

The cellular immune responses after immunization with the CZ171053 strain were evaluated by measuring cytokine production using the culture supernatants of stimulated splenocytes. As shown in Figure 4, although no significant production of IFN-γ or TNF-α was detected, IL-4 and IL-10 were efficiently produced.

### Intracellular survival determination

**Figure 5:**
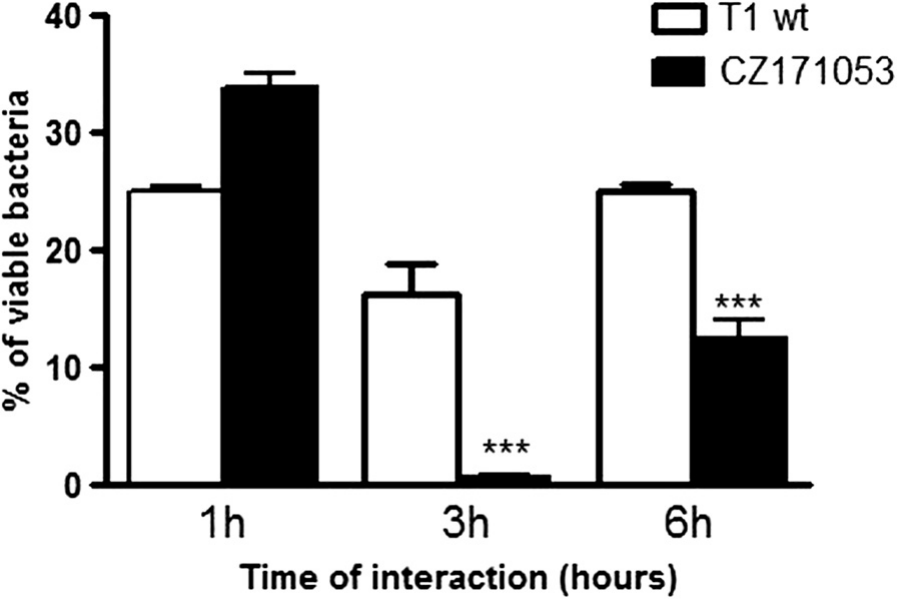
**Intracellular viability of the CZ171053 strain of *C. pseudotuberculosis *after one, three and six hours of interaction with J774 cells.** The values at each time-point represent the number of viable bacteria recovered relative to the number recovered at the previous time-point, expressed as a percentage. The results are expressed as the mean ± standard deviation of two representative experiments performed in quadruplicate. *** represents a significant reduction in viability relative to the T1 wild-type (wt) strain at *p* < 0.0001 (ANOVA, Tukey’s test).

The recombinant strain CZ171053 was submitted to intracellular viability analysis after one, three and six hours of interaction with a monolayer of J774 macrophage-like cells (Figure 5). We observed that after one hour of infection, the CZ171053 strain penetrated the cells more efficiently than the T1 wild-type strain. However, after three hours, the CZ171053 strain was essentially unable to survive inside the cells, suggesting that the mutation might impede its ability to persist inside the host. The analysis of the host-pathogen interaction after six hours revealed that the intracellular viability of the CZ171053 strain remained lower than with that of the T1 wild-type strain.

## Discussion

The results presented here are surprising because IFN-γ and TNF-α, which are important for the host defence against *C. pseudotuberculosis* infection [23,31], were not detected in significant levels in the immunized mice. We cannot exclude the possibility that this unexpected finding is due to the late time-point at which the cytokine levels were analysed. Simmons et al. [23] evaluated IFN-γ production on the seventh and fourteenth days after the vaccination of mice with live *C. pseudotuberculosis* and qualitatively detected the production of this cytokine on day 7, but not on day 14, suggesting a self-limiting profile of the immune response. The high production of IL-10 at a late time-point after inoculation was described by Paule et al. [32] and could be responsible for the low IFN-γ production; this observation suggests that an immune modulatory profile might underlie the inhibition of IFN- γ production observed in our experiments [33]. Additionally, this regulatory profile could be a consequence of an inflammatory reaction that led to the switch from a Th1 to a Th2 profile because cytokines typical of this profile were detected in our ELISA analyses. Furthermore, Paule et al. [32] observed that after caprine-immunization some animals did not produce IFN-γ but still presented significant protection against the infectious challenge. More accurate analyses should be performed to elucidate the immunological protection mechanisms that are induced by the CZ171053 strain in mice. In our experiments, cytokine production was evaluated 21 days after immunization, and the profile observed might be a modulatory profile after the initial immune response.

Our data on the immune protection, production of immunoglobulins and cytokines and intracellular viability suggest that the CZ171053 mutant has a reduced ability to survive inside the host cells but is still able to induce immunoglobulin and cytokine production. Furthermore, these results are suggestive of attenuation used by an iron-acquisition deficiency. Iron acquisition is essential for bacterial growth in vivo and the protein encoded by the gene that was disrupted in this mutant, *ciuA* (Gene ID: 9448933), is involved in the transport of iron from ferric citrate. The inability of this mutant to acquire iron might be important for attenuation. The mutant bacteria that invade the host cells are recognized by the immune system and, most importantly, protect the immunized mice upon challenge with a virulent strain. These results, which were obtained using a murine model, suggest that the CZ171053 strain may be used as a potential live vaccine against CLA in other animals such as goats and sheep, which are natural hosts for *C. pseudotuberculosis*.

## Competing interests

The authors declare that they have no competing interests.

## Authors’ contributions

DR performed the mouse experiments and wrote the manuscript, FSR performed the experiments with the J774 cells, KMCL was responsible for the immunoassays, SCS performed the statistical analysis, AS, RWDP, RM and SCO participated in the analysis and interpretation of the data, AM and VA made substantial contributions to the conception and design of this work, and FAD participated in the design and development of this study and also coordinated and helped draft this manuscript. All authors read and approved the final manuscript.
